# *Gardenia* Decoction Prevent Intestinal Mucosal Injury by Inhibiting Pro-inflammatory Cytokines and NF-κB Signaling

**DOI:** 10.3389/fphar.2019.00180

**Published:** 2019-03-28

**Authors:** Yizhe Cui, Qiuju Wang, Mengzhu Wang, Junfeng Jia, Rui Wu

**Affiliations:** College of Animal Science and Veterinary Medicine, Heilongjiang Bayi Agricultural University, Daqing, China

**Keywords:** *Gardenia jasminoides*, decoction, Lipopolysaccharide, cytokine, NfκB

## Abstract

*Gardenia jasminoides* Ellis, which belongs to the Rubiaceae family, is a widely used traditional Chinese medicine. Although effect of *Gardenia jasminoides* Ellis has been widely reported, its anti-inflammatory role in intestinal mucosal injury induced by LPS remains unclear. In the present study, we investigated the effects of decoction extracted from *Gardenia jasminoides* on the morphology and intestinal antioxidant capacity of duodenum induced by LPS in mice. Further analysis was carried out in the expression of inflammatory and anti-inflammatory cytokines. Nuclear factor-kappa B (NF-κB) was determined by Western blot. *Gardenia jasminoides* water extract was qualitative analyzed by high-performance liquid chromatography coupled with electro spray ionization quadrupole time-of-flight mass spectrometry. The results showed that *Gardenia* decoction markedly inhibited the LPS-induced Tumor necrosis factor (TNF)-α, Interleukin (IL)-6, IL-8, and IL-1 production. It was also observed that *Gardenia* decoction attenuated duodenum histopathology changes in the mouse models. Furthermore, *Gardenia* decoction inhibited the expression of NF-κB in LPS stimulated mouse duodenum. These results suggest that *Gardenia* decoction exerts an anti-inflammatory and antioxidant property by up-regulating the activities of the total antioxidant capacity (T-AOC), the total superoxide dismutase (T-SOD), and glutathione peroxidase (GSH-Px). *Gardenia* decoction is highly effective in inhibiting intestinal mucosal damage and may be a promising potential therapeutic reagent for intestinal mucosal damage treatment.

## Introduction

The intestine plays a crucial role in digesting and absorbing nutrients, balancing microbiota, protecting immunological functions and serves as a barrier against harmful pathogens and antigens (Vancamelbeke and Vermeire, 2017). Lipopolysaccharide (LPS/endotoxin), the major constituent of the outer membrane of gram-negative bacteria, is a common trigger of intestinal mucosal injury. The LPS-induced cytokine release leads to the pathophysiologic derangement associated with intestinal mucosal injury. Many cellular signals that are activated by Gram-negative bacteria are contributed to LPS. Not only does LPS trigger inflammatory responses, but it also activates pro-apoptotic signals in macrophages, endothelial cells, epithelial cells and immune cells (Ma et al., 2012; Plociennikowska et al., 2015). NF-κB plays a critical role in immune and inflammatory responses. It has been known to be present in most cell types and many of the inflammatory proteins expressed are regulated by NF-κB (Hayden and Ghosh, 2014).

Despite a growing understanding of the pathophysiology of intestinal mucosal damage, its molecular regulatory mechanisms in induction of cytokines expression and activation/recruitment of inflammatory in intestinal mucosal damage remain elusive. However, there is a need for innovative anti-inflammatory therapeutic protocols and some previous studies have shown that intestinal mucosal damage is associated with persistent activation of NF-κB (Medicherla et al., 2015). Therefore, the remedial measures of anti-inflammatory properties based on the NF-κB signaling pathway may be a potentially useful option. Likewise, growing evidence suggests that numerous components of Chinese medicinal herbs exert excellent anti-inflammatory effects through the negative regulation of NF-κB signaling pathway (Shen et al., 2018).

*Gardenia jasminoides*, an evergreen tree that belongs to the Rubiaceae family, is cultivated in multiple areas in China, with a Chinese name of Zhi Zi. It grows in many temperate regions and has fragrant white flowers (Ma et al., 2017). It is not only used as natural yellow dyes for many years (Chen et al., 2017; Ma et al., 2017), but also has various biological activities, such as antidiabetic (Wu et al., 2009), anti-inflammatory (Oliveira et al., 2017), anti-depression (Tao et al., 2014), and antioxidant properties (Guo et al., 2014), and improvement of the quality of sleep (Zhang et al., 2017). It is commonly used in traditional Chinese medicine. However, there are not so many reports studies focusing on the decoction of *Gardenia jasminoides*. Some studies showed that Geniposide inhibited Lipopolysaccharide-induced Apoptosis (Song et al., 2014). It is still not elucidated whether oral administration of *Gardenia jasminoides* decoction (GD) could provide a protective effect during intestinal mucosal injury and what is the underlying mechanism. The current study, we investigated the preventive effect of GD in LPS induced experimental intestinal mucosal injury in mice.

## Materials and Methods

### Reagents

The main reagents and antibodies used in our experiments are as follows: Antibodies recognizing NF-κB p65 (10745-1-AP) was purchased from Proteintech and β-actin was from Cell Signaling (Beverly, MA, USA). Secondary antibody was from Biosynthesis (Beijing, China). LPS from Escherichia coli 055:B5 was obtained from Sigma (St. Louis, MO, USA). LPS was suspended in physiological saline and stored as a 20 mg/ml stock. Animals were weighed before injecting LPS and the LPS of each animal was diluted to an appropriate dose. Dilute solution prior to injection were into normal saline.

### Animals

Animal protocols were approved by Heilongjiang Bayi Agricultural University's Institutional Animal Care and Use Committee. A total of 50 male ICR mice (22–25 g body weight) were purchased from the Animal Experiment Center of HARBIN MEDICAL UNIVERSITY (Daqing, China). All animals were kept in the temperature controlled room with 12 h dark/light cycles and maintained under specific-pathogen-free conditions and were given a standard mice diet and tap water for 1 week before experiments.

### Preparation of *G. jasminoides* Decoction

*Gardenia jasminoides* were purchased from Fu Rui Bang Chinese Medicine Co., Ltd. (Daqing, China). The general preparation procedure of *G. Jasminoides* decoction (GD) is as follows (Qin et al., 2015; Yu et al., 2015; Cui et al., 2017). Briefly, 100 g *G. jasminoides* Fruit were extracted by refluxing with water (1:10, w/v) for 2 h following sonication for 30 min, and then the extraction solutions were combined to be filtered and concentrated to 100 mL under reduced pressure. The concentrations of the residues were 1 g/mL for *G. jasminoides* fruit (Tao et al., 2014). Finally, the concentration be adjusted to the required with distilled water for intragastrical administration. After being autoclaved at 100°C for 20 min, the stock solution was stored at 4°C.

### LC/MS Analysis

The samples were thawed at room temperature, 100 μL of them was then transferred into Centrifuge Tubes (1.5 mL) by pipette. All samples were extracted with 300 μL of methanol, and 10 μL of internal standard (3 mg/mL, DL-o-Chlorophenylalanine) was added. The samples were then ultra-sonicated at 4 K Hz on ice bath for 30 min. The samples were vortexes for 30 s, and centrifuged at 12,000 rpm and 4°C for 15 min. Two hundred microliter of supernatant was transferred to vial for LC-MS analysis. Analysis platform: LC-MS (Thermo, Ultimate 3000LC, Orbitrap Elite) Column: Waters ACQUITYUPLC HSS T3column (2.1 mm × 100 mm, 1.8 μm) Chromatographic separation conditions: Column temperature: 40°C; Flow rate: 0.3 mL/min; Mobile phase A: water + 0.1% formic acid; Mobile phase B: acetonitrile + 0.1% formic acid; Injection volume: 4 μL; Automatic injector temperature: 4°C. The data was performed feature extraction and preprocessed with Compound Discoverer software (Thermo), and then normalized and edited into two-dimensional data matrix by excel 2010 software, including Retention time(RT), Compound Molecular Weight (comp MW), Observations (samples) and peak intensity.

### Grouping and Treatment

In experiments, animals were randomly divided into five groups: the normal control group, the LPS group, and GD high-dose, medium-dose and low-dose groups.GD-treated groups were given *G. jasminoides* decoction by intragastric administration once daily for 3 d. The normal control group and the LPS group were orally administered with double distilled water. One hour after the oral administration on 3 d, the control group received intraperitoneal injection of normal saline, while the other group received intraperitoneal injection of LPS (Escherichia coli 055:B5, 5 mg/kg; Sigma). At 20 h after the injection of LPS, all of the mice were sacrificed and their duodenum tissues were collected. The blood samples were centrifuged at 5,000 rpm for 10 min, and were subsequently stored at −80°C before analysis.

### Estimation of Cytokine Levels

Serum levels of various cytokines were estimated by enzyme-linked immunosorbent assay (Boster, Wu han, China). All analyses were conducted as described by the manufacturer.

### Duodenum Morphology

Part of the intestinal wall of the duodenum was prepared for histological examination by fixing in 4% formaldehyde-buffered solution, embedding in paraffin, and sectioning. The tissues were then embedded in paraffin and cut into 5 μm sections used for H&E staining (Cui et al., 2018). Villous height and the associated crypt depth were evaluated as described by Nabuurs et al. (1993) and Greig and Cowles (2017).

### Determination of Antioxidant Index in the Duodenum

To evaluate the provident-antioxidant balance in the duodenum, we determined total antioxidant capacity (T-AOC), the total superoxide dismutase (T-SOD), and glutathione peroxidase (GSH-Px) activities (Nanjing jiancheng Bioengineering institute). The method was describe as Fang et al. (2016). The duodenum samples were thawed, weighed, and homogenized (1:10, wt/vol) in 9 volumes of ice-cold physiologic saline. The homogenates were centrifuged at 3,000 × g for 10 min at 4°C, the supernatants collected and enzyme activities analyzed.

### Western Blot

The proteins were extracted from frozen intestinal tissues with an extract kit according to the manufacturer's protocol (cat. no. P0028; Beyotime Institute of Biotechnology, Haimen, China). Protein concentration was determined using a BCA assay. Equal amounts (50 μg per lane) of protein were subjected to 12% sodium dodecyl sulfate-polyacrylamide gel electrophoresis (SDS-PAGE) at 100 v for 3 h electrophoretic ally transferred to nitrocellulose/polyvinyl lidenedifluoride membranes (Pierce Biotechnology, Rockford, IL/Bio-Rad Laboratories, Hercules, CA), and blocked for 1 h in phosphatebuffered saline containing Tween 20 (0.1%) and non-fat milk (5%). The membranes were incubated overnight at 4°C with rabbit anti-mouse polyclonal antibodies to NF-κB (1:1,000 dilution), β-actin (1:2,000 dilution). After washing for three times with Tris-buffered saline containing Tween-20, the membranes were incubated with the corresponding goat anti-rabbit horseradish peroxidase-conjugated secondary antibody (1:10,000 dilutions) for 1 h at room temperature. Band intensities were measured using Image J software (National Institutes of Health).

### Data Analysis

All the experimental values obtained were expressed as mean ± SD. One-way ANOVA showed significant differences among multiple group comparisons. Analysis was performed with the software SPSS version 16.0 (SPSS Inc., USA). *P* < 0.05 was considered significant. Statistical significance was calculated by use of Student's *t*-test (two-group comparison).

## Results

### Characteristics of Compounds From the Herbal Formula GD

**Figure 1:**
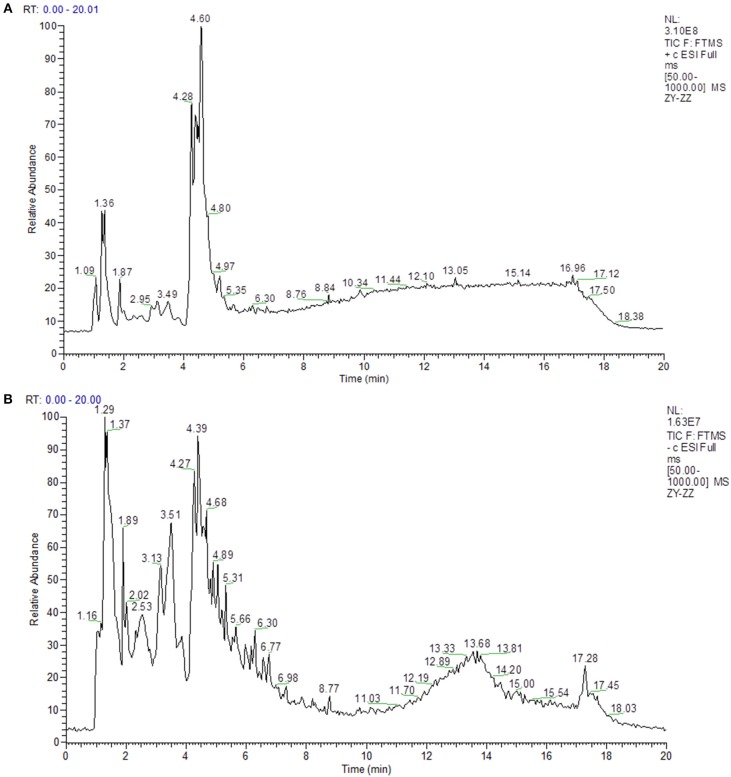
The Total Ion Chromatogram of GD. **(A)** The Total Ion Chromatogram of GD (ESI+). (ESI+) represents the positive ion detection mode, in which the mass analyzer scans only positive charged ions and filters out negative charged ions to obtain positive charged ions information during the detection process. **(B)** The Total Ion Chromatogram of GD (ESI–). (ESI–) denotes the negative ion detection mode, in which the mass analyzer scans only negative charged ions and filters out positive charged ions, thus obtaining the information of negative charged ions.

In this study, LC-MS analysis was performed in negative and positive ion modes to obtain complete information about the chemical constitution of GD. The peak MS spectrum has been presented in Figure 1. All constituents were full spectrum identified based on the accurate mass and network database Metlin. The identified compounds are shown in Tables 1,2.

**Table 1:** Chemical components identified from GD by high-performance liquid chromatography-electrospray ionization/mass spectrometry (ESI+).

**Name**	**Rt (min)**	**Molecular weight**	**CAS**	**Content (ng/μL)**
L-Phenylalanine	2.983	165.0785	63-91-2	79.79
L-Arginine	1.201	174.111	74-79-3	6.23
L-Tyrosine	1.397	181.0732	60-18-4	6.58
L-Glutamate	1.313	147.0525	56-86-0	1.72
L-Isoleucine	2.072	131.0941	61-90-5	28.07
L-Lysine	1.16	146.1049	56-87-1	0.21
L-Proline	1.341	115.0628	147-85-3	3.61
Pyroglutamic acid	1.98	129.042	98-79-3	7.89
Ferulic acid	4.69	194.0573	1135-24-6	18.63
Sinapic acid	4.66	224.0675	530-59-6	43.02
Styrene	6.038	104.0621	100-42-5	0.39
Chorismic acid	3.906	226.0474	617-12-9	4.24
m-Coumaric acid	4.64	164.0467	588-30-7	78.65
1,2,3-Trihydroxybenzene	3.232	126.0311	533-73-3	0.78
Caffeic Acid	4.48	180.0415	4607-41-4	3.03
Thymol	4.451	150.1038	89-83-8	15.89
Adenosine	1.965	267.0958	58-61-7	60.13
Adenine	1.957	135.0546	73-24-5	0.06
Guanosine	1.96	283.0908	118-00-3	3.02
Guanine	1.441	151.0487	73-40-5	1.98
cAMP	1.473	329.0502	60-92-4	11.12
Quercetin 3-galactoside	4.491	464.0946	482-36-0	3.94
Arcapillin	5.282	360.0832	NA	9.50
Glyceollin	5.928	338.1144	NA	0.02
Isorhamnetin	4.954	316.0571	480-19-3	0.65
Malvidin	5.267	330.0727	643-84-5	2.13
Naringenin	5.177	272.0675	480-41-1	0.07
Quercetin	4.988	302.0415	117-39-5	1.27
Quercetin 3-(3-p-coumaroylglucoside)	4.653	610.1301	76211-70-6	0.12
Rhamnetin	5.528	316.0572	480-19-3	0.03
Taxifolin	4.405	304.0572	480-18-2	0.09
Cyanidin 3-O-rutinoside	4.334	594.1558	28338-59-2	0.29
Diosmetin	5.179	300.0621	520-34-3	0.51
Eriodictyol	4.51	288.0621	552-58-9	0.11
Genistein	4.478	270.0516	446-72-0	0.15
Genistin	4.476	432.1037	529-59-9	0.30
Luteolin	4.806	286.0465	491-70-3	1.53
Pelargonidin 3-O-(6-O-malonyl-β-D-glucoside)	4.525	518.1035	165070-68-8	0.08
Pelargonidin 3-O-rutinoside	4.389	578.1612	NA	0.72
Petunidin 3-O-glucoside	4.537	478.1092	6988-81-4	0.09
Quercitrin	4.503	448.0987	522-12-3	1.07
Sakuranin	4.532	448.1351	NA	0.10
Scutellarein 5-glucuronide	4.501	462.0778	NA	0.25
Naringin	4.482	580.1763	10236-47-2	0.64
Gallocatechin	1.388	306.0707	NA	4.92
Peonidin 3-rhamnoside 5-glucoside	13.76	609.1748	53859-11-3	0.26
Hesperetin	4.538	302.0778	520-33-2	0.58
2-Hexyl-3-phenyl-2-propenal	5.773	216.1506	101-86-0	0.98
DL-pipecolic acid	1.925	129.0785	535-75-1	3.26
Hydroquinidine	4.963	326.1984	1435-55-8	0.03
Hypoxanthine	1.963	136.0379	68-94-0	0.25
Trigonelline	1.584	137.0471	535-83-1	0.63
Xanthosine	4.474	284.0787	146-80-5	0.20
Caffeine	4.413	194.0837	58-08-2	0.10
D-Mannitol	1.231	182.0785	69-65-8	0.72
a-L-Rhamnose	1.239	164.0679	6014-42-2	0.83
Gibberellin A53	5.402	348.1923	NA	0.08
Glutinosone	5.699	220.1455	55051-94-0	0.37
Plaunol B	4.789	356.1247	69749-00-4	2.28
Quillaic acid	6.58	486.3329	631-01-6	8.02
Genipin	4.406	226.083	6902-77-8	12.45
Medicagenic acid	6.215	502.327	599-07-5	5.55
p-Cymene	4.894	134.1089	NA	0.56
Pantothenic Acid	3.524	219.1103	137-08-6	59.57
Pyridoxine	2.326	169.0736	65-23-6	1.04
Pyridoxal	3.258	167.0579	66-72-8	0.09
Niacin	5.633	123.0314	59-67-6	0.09
Niacinamide	1.985	122.0473	98-92-0	7.07
Palmitic amide	9.57	255.2558	629-54-9	8.89
13Z-Docosenamide	13.06	337.3334	112-84-5	20.22
Oleamide	9.873	281.2709	301-02-0	34.54
Stearamide	12.982	283.2865	124-26-5	1.67
Coumarin	5.111	146.0362	91-64-5	1.58
3 Hydroxycoumarin	3.902	162.0309	939-19-5	10.58
Scopoletin	4.766	192.0414	NA	3.25
Benzoic acid	4.7	122.0362	65-85-0	0.50
α-ketoisovaleric acid	1.86	116.0469	759-05-7	1.07
Succinic acid	1.957	118.0273	110-15-6	14.87
nandrolone	5.468	274.1923	434-22-0	1.36
α-Linolenic Acid	7.357	278.224	463-40-1	2.15
Butyric acid	1.866	88.0521	107-92-6	2.26
LysoPC (16:0)	7.257	495.3313	NA	9.12
MG (0:0/18:3/0:0)	6.214	352.2602	NA	1.28
Indoleacrylic acid	4.278	187.0625	1204-06-4	25.29
Methyl cinnamate	3.805	162.0675	103-26-4	15.33
5-Hydroxy-L-tryptophan	2.276	220.0845	4350-09-8	7.11
Indoleacetaldehyde	2.371	159.0681	NA	0.98
Acetylcholine	2.005	145.1099	51-84-3	0.04
Cinnamic acid	3.612	148.0521	621-82-9	5.94
Gingerol	5.765	294.182	58253-27-3	1.93
Hippuric acid	4.356	179.0576	495-69-2	0.28
Jasmolone	5.898	180.1144	54383-66-3	5.56
(-)-Jasmonic acid	5.713	210.1247	6894-38-8	0.04
Indole	4.301	117.0573	120-72-9	19.62
Methyl jasmonate	4.519	224.1403	39924-52-2	12.53
Phenylacetic acid	4.746	136.0518	103-82-2	2.22
Acetophenone	4.403	120.0568	98-86-2	148.13
Choline	9.289	103.0991	62-49-7	2.23
Tropic acid	4.458	166.065	552-63-6	0.28

**Table 2:** Chemical components identified from GD by high-performance liquid chromatography-electrospray ionization/mass spectrometry (ESI–).

**Name**	**Rt (min)**	**Molecular weight**	**CAS**	**Content (ng/μL)**
L-Isoleucine	2.06	131.09469	61-90-5	69.85
L-Phenylalanine	2.933	165.07893	63-91-2	1577.95
Pyroglutamic acid	1.991	129.04272	98-79-3	130.58
L-Cystine	4.179	240.02653	56-89-3	29.79
Chlorogenic Acid	4.127	354.09478	327-97-9	6.83
ferulic acid	4.705	194.0574	1135-24-6	67.37
Sinapic acid	4.68	224.06787	530-59-6	1442.50
1,2,3-Trihydroxybenzene	3.154	126.03172	533-73-3	406.95
Caffeic Acid	3.013	180.04208	4607-41-4	13.88
Gallic acid	3.708	170.02138	149-91-7	75.26
Gentisic acid	3.623	154.0266	490-79-9	418.77
Shikimic acid	1.836	174.05273	138-59-0	1125.65
Homogentisic acid	3.694	168.04204	451-13-8	92.70
m-Coumaric acid	4.65	164.04712	588-30-7	85.87
Syringic acid	2.887	198.05249	530-57-4	259.47
Salicylic acid	4.496	138.03141	69-72-7	186.01
Uridine	2.02	244.06907	58-96-8	29.12
Inosine	1.276	268.07889	58-63-9	1744.91
IMP	4.452	348.04661	131-99-7	5.67
cAMP	1.971	329.05183	60-92-4	12.55
Diosmetin	5.179	300.06245	520-34-3	10.54
Genistein	4.566	270.05208	446-72-0	5.29
Malvidin	5.272	330.07307	643-84-5	49.08
Naringenin	5.185	272.06776	480-41-1	5.08
Quercetin	5.038	302.04179	117-39-5	151.07
Cyanidin 3-O-rutinoside	4.326	594.15626	28338-59-2	215.56
Isorhamnetin	4.948	316.05741	480-19-3	28.09
Luteolin	4.861	286.04682	491-70-3	98.57
Pelargonidin 3-O-rutinoside	4.944	578.16133	NA	7.12
Petunidin 3-O-glucoside	4.585	478.10942	6988-81-4	12.44
Quercitrin	4.555	448.09913	522-12-3	54.73
Dihydromyricetin	4.479	320.05192	27200-12-0	5.70
Eriodictyol	4.523	288.06209	552-58-9	6.25
Naringin	4.499	580.17667	10236-47-2	497.90
Quercetin 3-(3-p-coumaroylglucoside)	4.67	610.12941	76211-70-6	5.09
Quercetin 3-galactoside	4.519	464.09335	482-36-0	355.12
Scutellarein 5-glucuronide	4.502	462.07786	NA	26.79
Taxifolin	4.43	304.05702	480-18-2	5.79
Rutin	4.428	610.14931	153-18-4	869.84
Hesperetin	4.523	302.07789	520-33-2	7.26
Purine	1.299	120.04223	120-73-0	2218.84
2-Furoic acid	1.439	112.01615	88-14-2	5378.42
Caffeine	4.492	194.08423	58-08-2	8.44
D-Glucarate	1.543	210.03737	87-73-0	1339.49
D-Glucuronic acid	1.264	194.04247	6556-12-3	465.85
Glutaric acid	1.311	132.04226	110-94-1	14780.91
L-Xylulose	1.458	150.05294	527-50-4	77.69
D-Mannitol	1.265	182.07878	69-65-8	3230.88
Gluconic acid	1.299	196.058	526-95-4	15723.38
α-D-Glucose	1.307	180.06317	492-62-6	2610.07
α,α-Trehalose	1.738	342.1154	57-50-1	694.30
Raffinose	4.067	504.16731	512-69-6	9.04
Genipin	4.414	226.08368	6902-77-8	1182.88
Gibberellin A12	8.093	332.19787	NA	7.66
Medicagenic acid	6.193	502.32825	599-07-5	5112.01
Quillaic acid	6.564	486.33328	631-01-6	2459.37
Rishitin	7.443	222.16141	18178-54-6	495.43
Gibberellin A17	4.924	378.1664	18411-79-5	48.05
Gibberellin A36	5.465	362.17181	NA	56.04
Ganoderic acid H	17.348	572.2945	98665-19-1	391.13
Geranyl diphosphate	4.391	314.06284	763-10-0	9.75
Pantothenic Acid	3.485	219.1103	137-08-6	1709.46
Riboflavin	4.246	376.1359	83-88-5	416.48
Sulfuric acid	1.575	97.96744	7664-93-9	5308.81
Phosphoric acid	1.471	97.97696	7664-38-2	473.05
Benzoic acid	4.717	122.03673	65-85-0	171.79
Citric acid	1.446	192.02674	77-92-9	56678.22
Lactic acid	2.959	90.0318	50-21-5	37.83
Pyruvate	1.45	88.01615	127-17-3	1368.82
Hexadecanedioic acid	5.656	286.21382	NA	37.39
Quinic acid	4.373	192.06302	77-95-2	1839.95
Aconitic acid	2	174.0164	499-12-7	1098.77
Itaconic acid	2.512	130.02669	97-65-4	344.08
Maleic acid	1.996	116.01102	110-16-7	192.83
Malic acid	1.879	134.02155	6915-15-7	13050.35
Oxoglutaric acid	1.487	146.02162	328-50-7	1208.40
Succinic acid	2.072	118.02664	110-15-6	8700.38
Glyceric acid	1.354	106.02678	473-81-4	223.93
Nandrolone	5.46	274.19264	434-22-0	7.66
α-Linolenic Acid	7.321	278.22397	463-40-1	140.85
LysoPC(15:0)	7.22	481.31539	NA	978.13
Traumatic Acid	5.273	228.13561	6402-36-4	66.26
Acetophenone	4.646	120.05742	98-86-2	42.39
Citramalic acid	1.499	148.03727	2306-22-1	1513.97
Mevalonic acid	3.028	148.07363	150-97-0	44.67
Phenylacetic acid	4.741	136.05243	103-82-2	150.15
(-)-Jasmonic acid	5.711	210.12533	6894-38-8	5.62
Malonic acid	1.474	104.0111	141-82-2	304.15
Xanthoxin	6.275	250.15644	8066-07-07	8.61
Gentisin	4.621	258.05214	437-50-3	38.04
Tropic acid	4.432	166.06257	552-63-6	341.15
Xanthoxic acid	9.992	266.15443	NA	39.28

### Serum Concentrations of Cytokine

**Figure 2:**
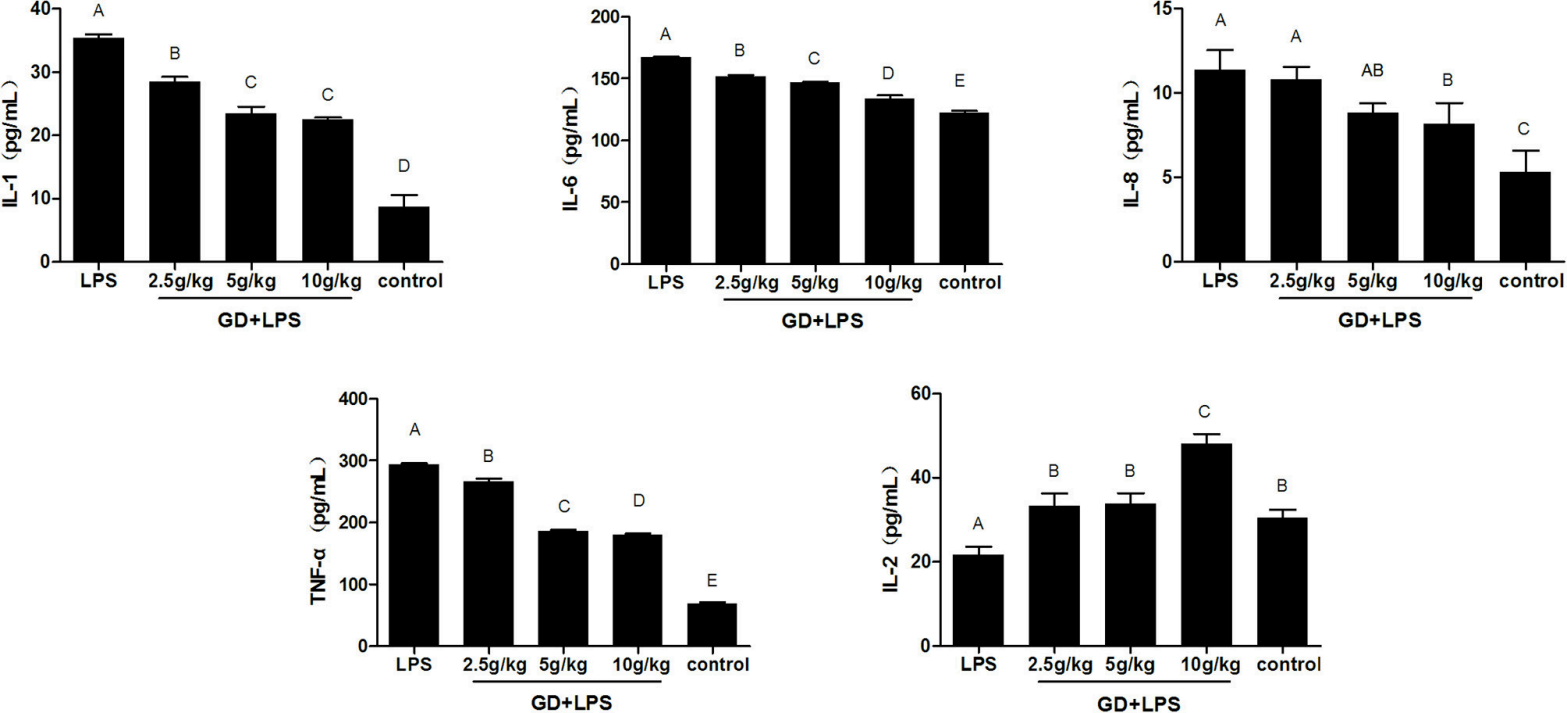
Effects of GD on the production of inflammatory cytokines in the serum. The data are expressed as the mean ± SD (*n* = 10 per treatment group). The values with same superscript letters between groups are of no significant difference (*P* > 0.05), those with same letters are of significant difference (*P* < 0.05). Interleukin (IL)−1, IL-2, IL-6, IL-8, and Tumor necrosis factor (TNF)-α.

**Figure 3:**
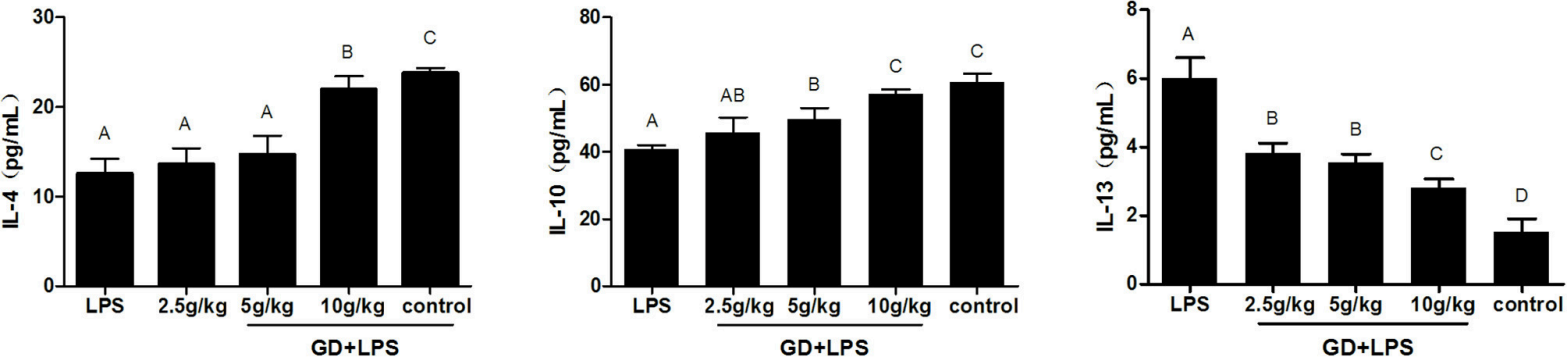
Effects of GD on the production of anti-inflammatory cytokines in the serum. The data are expressed as the mean ± SD (*n* = 10 per treatment group). The values with same superscript letters between groups are of no significant difference (*P* > 0.05), those with different letters are of significant difference (*P* < 0.05). Interleukin (IL)−4, IL-10, and IL-13.

Compared with the control group, the concentration of inflammatory cytokine IL-1, IL-6, IL-8, and TNF-α in the LPS model group were significantly higher (*P* < 0.05). The level of IL-1, IL-6, IL-8, and TNF-α in the low, medium and high dose GD-treated group were significantly lower than that of the LPS group with a dose-dependent manner (*P* < 0.05). IL-2 was contrary to the changes of other inflammatory cytokines (Figure 2). In addition, compared with the control group, the concentration of anti-inflammatory cytokine IL-4, and IL-10 were significantly reduced in the LPS model group in the serum (*P* < 0.05). Compared with the LPS model group, the level of IL-4 and IL-10 in the low, medium and high dose GD-treated group were gradually increased with a dose-dependent manner and significant difference (*P* < 0.05). However, IL-13 was contrary to the changes of other anti-inflammatory cytokines (Figure 3).

### Histopathological Changes in Duodenum Tissue

**Figure 4:**
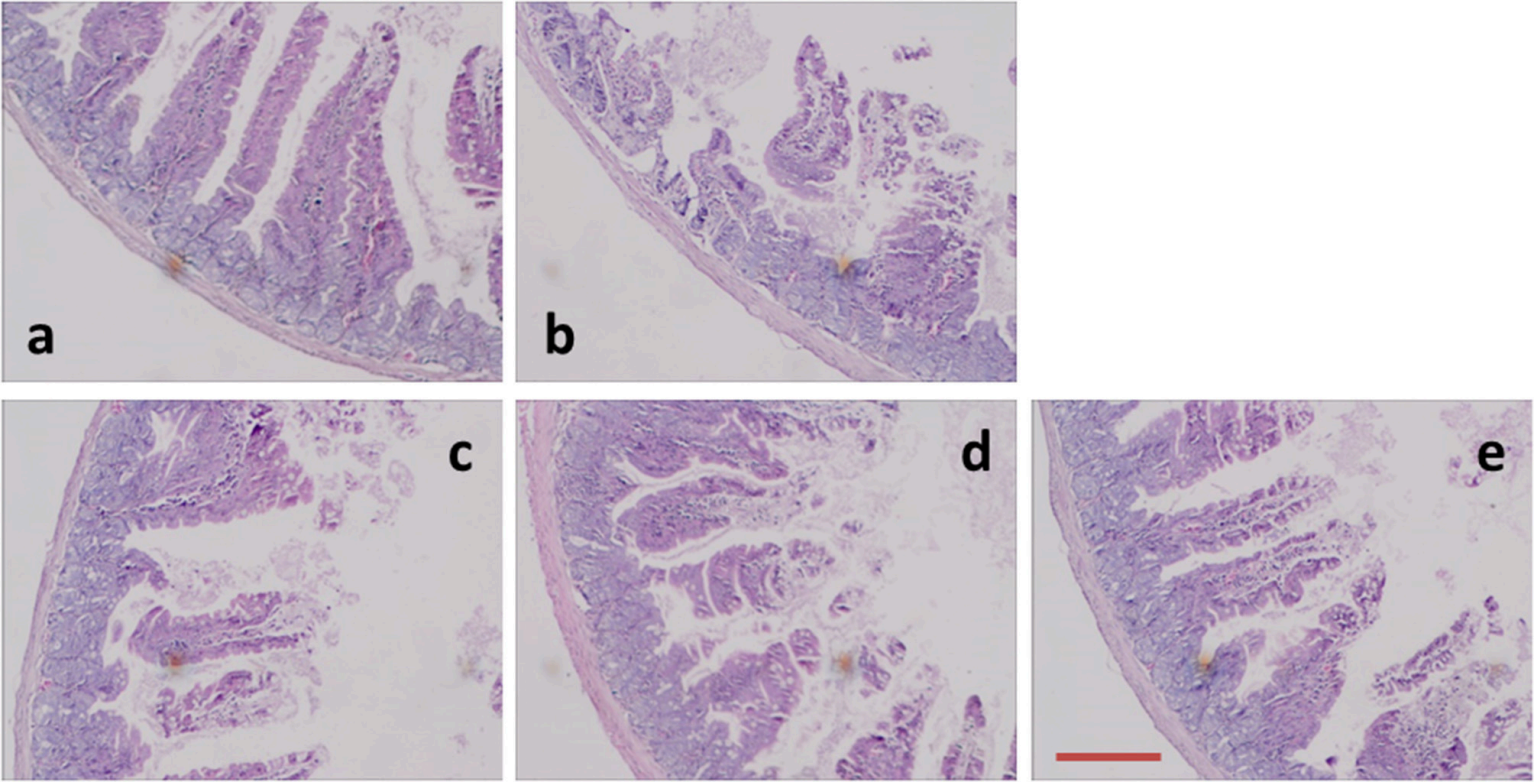
Photomicrographs of mice duodenum tissues. **(a)** Control group, **(b)** LPS group, **(c)** GD low-dose group, **(d)** GD medium-dose group, and **(e)** GD high-dose group. Histological appearance of mice intestinal mucosa after hematoxylin and eosin (H&E) stain (original magnification 100×). Scale bars: 50 μm.

The microscopic morphology was observed with HE staining. The pathological changes were obvious in the duodenum (Figure 4). Compared to the control animals, LPS-treated groups caused significant mucosal damage, and that is, epithelial shedding, villi fracturing, mucosal atrophy, edema and the villus had shortened. The length of duodenal villi in GD medium and high dose groups increased significantly compared with that in LPS group (*P* < 0.05), and the degree of intestinal mucosal injury was significantly lower than that in LPS group. Under the light microscope, the degree of duodenal mucosal injury was graded according to the standard (Chiu et al., 1970). The score of intestinal mucosal injury in each group was shown in Table 3. Compared with LPS group, the damaged level of intestinal mucous membrane was slighter than that of medium and high dose groups.

**Table 3:** Effects of GD on the morphological structure of duodenum in the mice.

**Groups**	**VH**	**CD**	**V/C**	**Mucosal injury**
LPS	151.34 ± 15.26^A^	74.75 ± 7.42^A^	2.32 ± 0.52^A^	4.32 ± 0.42^A^
Low	175.25 ± 19.07^A^	62.88 ± 12.30^AB^	2.56 ± 0.70^A^	3.97 ± 0.34^A^
medium	213.00 ± 29.91^B^	60.38 ± 6.22^AB^	3.46 ± 1.46^AB^	2.65 ± 0.24^B^
High	246.5 ± 16.62^B^	54.12 ± 3.25^B^	4.36 ± 0.15^BC^	1.23 ± 0.28^C^
Control	272.53 ± 24.65^B^	53.75 ± 2.84^B^	5.47 ± 0.78^C^	0.54 ± 0.02^D^

*The data are expressed as the mean ± SD (n = 10 per treatment group). VH (villus height), CD (crypt depth), and V/C (villus height/crypt depth). Grade of intestinal mucosal injury. Grade 0, normal mucosa; Grade 5, the most extensive denudation of mucosa. The difference between groups was significantly different in different capitals*.

### Histomorphological Analyses

As shown in Table 3, compared with the normal group, the Villus height (VH) and ratio of villus height to crypt depth (V/C) decreased significantly in LPS group. In addition, the VH decreased significantly in low dose group. The crypt depth (CD) was higher in the GD treatment group than that of control group, but lower than that of LPS group. The VH in high dose group decreased slightly, and the CD and V/C were not significantly different from those in normal group, which was significantly higher than that in LPS group.

### Antioxidant Indicators in the Duodenum

**Figure 5:**
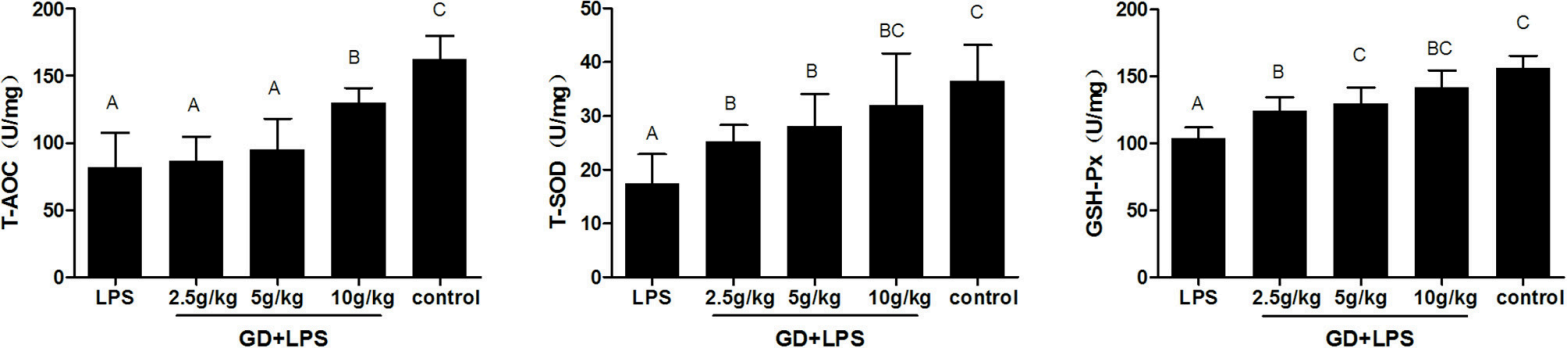
Effects of GD on the antioxidant status of duodenum in the mice. The data are expressed as the mean ± SD (*n* = 10 per treatment group). The total superoxide dismutase (T-SOD), total antioxidant capacity (T-AOC), glutathione peroxidase (GSH-Px). The values with same letters between groups are of no significant difference (*P* > 0.05), those with different letters are of significant difference (*P* < 0.05).

As the Figure 5 shows, compared with the control, the activities of the T-AOC, T-SOD, and GSH-Px significantly decreased in the LPS and low dose group, however, there was no significantly difference in high dose group. In addition, the content of T-AOC, T-SOD, and GSH-Px in each GD treatment group was higher than that in the LPS group. Moreover, in the process of the dose increased, the enzyme activity was significantly increased.

### Effects of GD on NF-κB Expression

**Figure 6:**
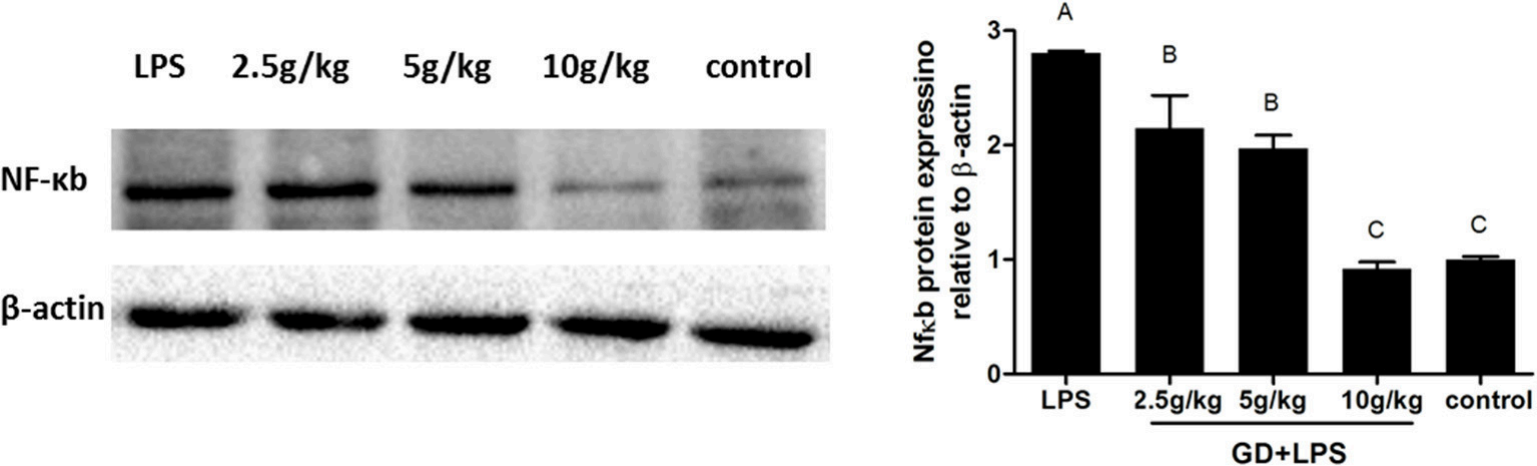
Effects of GD on NF-κB signaling pathway activation. Western blot analysis was used to assess the expression of NF-κB from each group and quantitated protein band intensities presented as β-actin normalized mean values. Representative western blot images and quantified expression levels are presented. Values are expressed as the mean ± standard deviation (*n* = 10). The values with same letters between groups are of no significant difference (*P* > 0.05), those with different letters are of significant difference (*P* < 0.05). NF-κB, nuclear factor-κB.

To evaluate the effects of GD on expression of NF-κB, the level of NF-κB in duodenum were assayed (Figure 6). In the GD treatment group and control group, the expression of NF-κB was lower significantly (*P* < 0.05) than in LPS group. In addition, in the GD low and medium dose groups the expression of NF-κB was higher significantly (*P* < 0.05) than in control group, however, there was no significantly difference in high dose group. In our present study, Western blot analysis revealed that GD can modify NF-κB activity.

## Discussion

Complete function of intestinal mucosa is essential for health and survival (Liu et al., 2008). As the main mediator between pathogen and intestinal tract, intestinal epithelial cells play an important role in the defense of pathogens (Qian et al., 2016). The function of intestinal epithelial cells depends on the homeostasis of intestinal mucosa. There is growing evidence that the balance between intestinal epithelial cells and the immune system maintains intestinal health (Maloy and Powrie, 2011). We studied whether GD could improve LPS-induced inflammation in mice. In the current experiment, we employed LPS as an inflammatory agent to establish a model of intestinal injury in mice. LPS challenge increased the level of TNF-α, IL-1, IL-6, and IL-8 in the serum (Figure 2). Importantly, GD reduced the concentrations of TNF-α, IL-1, IL-6, and IL-8 in the serum, compared to LPS-challenged mice. These findings indicate that the GD has beneficial effects in reducing intestinal mucosal inflammation. The level of IL-2 has a positive correlation with cellular immune function, and it can also improve the immune defense and immune repair ability of cells. Many studies have shown that the decrease of IL-2 can lead to cellular immune dysfunction (Boyman et al., 2015). In the GD treatment groups the level of IL-2 was significantly enhanced, indicating that the level of IL-2 increased the immune function of T cells, thus inhibiting the inflammation in the intestine. IL-10 has multiple functions such as immunomodulation and anti-inflammatory effects (da Silva et al., 2015). In this study, the expression level of IL 10 in the serum of the model group mice was lower than that of the control group, indicating that the expression level of IL-10 was negatively correlated with the degree of inflammation. The GD played a significant role in regulating IL-10 level, and increased with the increase of GD concentration (Figure 3). Importantly, significant correlation between NF-κB activity and concentrations of pro-inflammatory mediators was revealed in intestine (Zuo et al., 2013). The activation of NF-κB leads to production of pro-inflammatory molecules. Previous studies show that *Gardenia jasminoides* Ellis and Crocus sativus L could decrease NF-κB and inflammation (Xu et al., 2009). Our results showed that LPS significantly increased the expression of NF-κB, while blockade of GD significantly abolished these effects (Figure 5). GD significantly regulates the imbalance between pro-inflammatory and anti-inflammatory factors in the duodenum tissue of mice, down regulates the state of local immunoreaction and alleviates the damage of mucosal inflammation, which may be one of the mechanisms for the treatment of intestinal mucosal damage.

The complete structure of the small intestine is the physiological basis of its digestion and absorption function, and its morphological and structural changes directly affect the surface area of villi, thereby affecting the body's ability to absorb nutrients (Collins and Bhimji, 2017). VH, CD, and V/C can be regarded as a criterion to reflect the intestinal mucosal morphology and the absorption capacity of the small intestine (Greig and Cowles, 2017). VH and CD of intestinal mucosa are closely related to animal digestion. Detection of VH and CD can judge the degree of intestinal mucosal damage and the ability to repair (Dong et al., 2016). Thus, an increase in VH, V/C or decrease in the CD corresponds to an improvement in the digestion and absorption of nutrients (Hou et al., 2013). Accordingly, GD increased V/C and VH in the duodenum and decreased the VH, compared to the LPS mice. The result of serum metabolites (Figure 1) was also in agreement with the alteration of intestinal villus structure. These results indicated that GD has inhibitory effect on the intestinal mucosal damage in mice, and the inhibitory effect exhibits a dose-dependent manner.

HPLC analysis identified the main components-amino acids, organic acids, fatty acids, nucleosides, flavonoids and so on-included in GD. Geniposide, the major iridoid glycoside ingredient of gardenia herbs, has emerged as a novel multifunctional tissue-protective agent with antioxidant (Fu et al., 2012) and anti-inflammatory effects (Lee et al., 2009). SOD is an important enzyme system for scavenging oxygen free radicals, which has protective effect on cell damage. SOD can prevent the expansion of oxidation free radical chain reaction. It can be considered as an important line of oxygen free radical scavenging system in organism (Mansuroglu et al., 2015). GSH-PX catalyzes the redox reaction of the prototype GSH to the hydroperoxide, which can remove the harmful peroxide metabolites in the cells and block the lipid peroxidation chain reaction, thus protecting the membrane structure and function integrity of the cell (Gordeeva et al., 2015). The results of the experiment were that the content of T-AOC in GD treatment groups was higher than that of the model group, and there was a significant difference. The expression of T-SOD and GSH-Px in the GD treatment groups was significantly increased in the duodenum tissue and there was a significant difference (Figure 6). It is indicates that GD has the ability to protect the intestinal epithelial cells from oxygen free radical damage.

## Conclusion

*Gardenia jasminoides* can promote tissue repair by inhibiting the expression of inflammatory factors, lowering the disease activity and deceasing intestinal mucosal damage. It could be important for intervening the cycle of inflammation associated with intestinal mucosal injury. Further studies of GD are necessary to develop a new effective plant-derived therapeutic modality for intestinal mucosal injury.

## Author Contributions

YC and QW designated the study, collected and analyzed the data, and wrote the manuscript. MW and JJ contributed to data collection. RW supervised the study. All authors reviewed and approved the manuscript.

### Conflict of Interest Statement

The authors declare that the research was conducted in the absence of any commercial or financial relationships that could be construed as a potential conflict of interest.
